# TLE3 as a candidate biomarker of response to taxane therapy

**DOI:** 10.1186/bcr2241

**Published:** 2009-03-23

**Authors:** Swati A Kulkarni, David G Hicks, Nancy L Watroba, Christine Murekeyisoni, Helena Hwang, Thaer Khoury, Rodney A Beck, Brian Z Ring, Noel C Estopinal, Marshall T Schreeder, Robert S Seitz, Douglas T Ross

**Affiliations:** 1 Department of Surgical Oncology, Roswell Park Cancer Institute, Elm and Carlton Streets, Buffalo, NY 14263, USA; 2 Department of Pathology and Laboratory Medicine, University of Rochester, 601 Elmwood Avenue, Rochester, NY 14642, USA; 3 Department of Pathology, Roswell Park Cancer Institute, Elm and Carlton Streets, Buffalo, NY 14263, USA; 4 Applied Genomics Inc., 601 Genome Way, Huntsville, AL 35806, USA; 5 Applied Genomics Inc., 863 Mitten Road #103, Burlingame, CA 94010, USA; 6 Department of Radiation Oncology, Center for Cancer Care, Suite 10, 201 Sivley Road SW, Huntsville, AL 35805, USA; 7 Department of Medical Oncology, Clearview Cancer Institute, 3601 CCI Drive, Huntsville, AL 35805, USA

## Abstract

**Introduction:**

The addition of taxanes (Ts) to chemotherapeutic regimens has not demonstrated a consistent benefit in early-stage breast cancer. To date, no clinically relevant biomarkers that predict T response have been identified.

**Methods:**

A dataset of immunohistochemistry stains in 411 patients was mined to identify potential markers of response. TLE3 emerged as a candidate marker for T response. To test the association with T sensitivity, an independent 'triple-negative' (TN) validation cohort was stained with anti-TLE3 antibody.

**Results:**

TLE3 staining was associated with improved 5-year disease-free interval (DFI) in the overall cohort (n = 441, *P *< 0.004), in patients treated with cyclophosphamide (C), methotrexate, and 5-fluorouracil (n = 72, *P *< 0.02), and in those treated with regimens containing doxorubicin (A) and a T (n = 65, *P *< 0.04). However, no association was shown with outcome in untreated patients (n = 203, *P *= 0.49) or those treated with a regimen containing A only (n = 66, *P *= 0.97). In the TN cohort, TLE3 staining was significantly associated with improved 5-year DFI in all patients (n = 81, *P *< 0.015), in patients treated with AC + T (n = 45, *P *< 0.02), but not in patients treated with AC (n = 17, *P *= 0.81). TLE3 was independent of tumor size, nodal status, and grade by bivariable analysis in both cohorts.

**Conclusions:**

TLE3 staining is associated with improved DFI in T-treated patients in two independent cohorts. Since the validation study was performed in a TN cohort, TLE3 is not serving as a surrogate for estrogen receptor or HER2 expression. TLE3 should be studied in large clinical trial cohorts to establish its role in T chemotherapy selection.

## Introduction

Breast cancer is a disease that shows significant biologic diversity and a spectrum of clinical behaviors with important differences in response to therapy. The application of molecular profiling to patient samples and the resultant evolving molecular classification of breast cancer have identified at least five subtypes, which can be distinguished by characteristic gene expression profiles: two luminal subsets within estrogen receptor (ER)-expressing tumors and three groups within mostly ER^- ^tumors (HER2, normal breast-like, and the basal-like subtypes) [1]. In the clinical literature, the immunohistochemically defined 'triple-negative' (TN) (ER, progesterone receptor (PR), and HER2) class has generated considerable interest given their poor prognosis, an association with hereditary tumors, and the lack of established therapies that target this subtype of breast cancer.

Numerous clinical trials and a large meta-analysis have demonstrated a survival benefit from adjuvant chemotherapy in women with breast cancer [2]. The taxanes, including paclitaxel and docetaxel, are among the most active agents available [3,4]. The mechanism of action of these drugs is related in part to the stabilization of microtubules and the induction of G_2_/M arrest, with subsequent apoptosis of tumor cells [5,6]. These agents have become the standard of care for first-line treatment of metastatic breast cancer and are frequently incorporated into both adjuvant and neoadjuvant anthracycline-containing regimens. However, the addition of taxanes to cytotoxic regimens has not always demonstrated a consistent improvement in outcomes, particularly in early-stage breast cancer [7]. The variable benefit seen from taxane therapy is likely the result of the heterogeneous nature of breast cancer.

We have endeavored to translate the gene expression-based classification of carcinoma into immunohistochemistry (IHC) reagents that can be used to discover and validate the relationship between tumor classification and clinically significant phenotypes [8]. Using gene expression data to target our efforts, we have generated over 700 novel rabbit antisera and screened through these and hundreds of commercially available antisera to identify those with utility in classifying breast cancer. We have developed sets of antibodies termed 'panels of diversity' which classify the biologic diversity of carcinoma, and we have now focused on using these panels to discover single or multiple reagents that can be combined using multivariate index assays to predict outcome for defined clinical applications. We describe herein the nomination and validation of TLE3 as a novel biomarker of response to taxane therapy in breast cancer. TLE3 is a member of the transducin-like enhancer of split (TLE) family of proteins that have been implicated in the tumorgenesis and classification of sarcomas [9,10]. It is a transcriptional repressor homologous to drosophila groucho proteins involved in repressing epithelial cell fate determination [11,12]. It interacts with the Notch/WNT pathway and appears to be periodically expressed during the M phase of the cell cycle [13,14]. We validate its association with outcome in taxane-treated patients using a qualitative IHC test in a cohort of TN breast cancer patients.

## Materials and methods

### Patient samples and assembly of clinical datasets

Institutional breast cancer cohorts from the Clearview Cancer Institute of Huntsville (CCIH) and the Roswell Park Cancer Institute (RPCI) were used in this study. In all cohorts, patient tumor paraffin blocks were assigned an anonymous unique identifier linked to clinical databases that contained treatment and outcome data. Institutional review board approval was obtained for the use of patient blocks at each respective institute. A previously assembled dataset of IHC stains in 411 patients from the CCIH cohort diagnosed between 1989 and 2002 was mined to identify biomarkers of chemotherapy response [8]. The RPCI Breast Cancer Database used for the validation study contains all patients diagnosed with breast cancer and treated with surgery at RPCI from January 1996 to January 2006. Cases are entered into the database consecutively as they are seen and treated. Eighty-one ER^-^, PR^-^, and HER2^- ^surgical cases were identified and paraffin blocks with adequate tissue for analysis by tissue microarray (TMA) were retrieved from the pathology archives. Patients who received adjuvant and/or neoadjuvant chemotherapy were included. For those patients who received neoadjuvant chemotherapy and had a complete pathologic response (pCR) (no residual tumor identified in the breast and axillary surgical specimens), the diagnostic core biopsy specimen was stained without incorporation into a TMA. Patients were excluded from the study if their core biopsy was performed at an outside institution and showed a pCR (no tissue available at RPCI for analysis). The TN immunophenotype for all cases was confirmed by re-staining at RPCI. The clinical and pathologic characteristics of the discovery, validation, and neoadjuvant cohorts were extracted from the clinical records by chart review (Tables 1 and 2). When the data mining of the CCIH clinical dataset was conducted, any adjuvant chemotherapy regimen that contained both doxorubicin (A) and a taxane (T) (either paclitaxel or docetaxel) was compared with any regimen containing A without T. The prospectively designed validation study was limited to comparing patients who received doxorubicin and cyclophosphomide (AC) with those who received AC + T (Table 3). Response to therapy in both the discovery and validation cohorts was assessed by the absence of local or distant recurrence and by the absence of new contralateral breast cancer based on negative imaging studies and clinical examination. In the neoadjuvant cohort, pCR was determined by review of pathology reports that demonstrated no viable tumor in the breast or in the axilla. Pre-treatment tumor size and lymph node status were determined by chart review of the physical exam findings and imaging reports. When imaging reports were not available, archived films were reviewed with the RPCI staff mammographer. Magnetic resonance imaging (MRI) was not used extensively prior to 2003 at RPCI, but when available, tumor size based on MRI findings was used to determine the pre-treatment size of the primary tumor. If there was a discrepancy between tumor size based upon physical examination and imaging, physical examination was used to determine pre-treatment stage.

**Table 1:** Clinical and pathologic characteristics of the Clearview Cancer Institute in Huntsville discovery cohort and the Roswell Park Cancer Institute validation cohort

		CCIH		RPCI	
		Number	Percentage	Number	Percentage
Total patients		411	100	81	100
Age	< 50 years	117	28	33	41
	≥ 50 years	294	72	47	58
	Unknown	0	0	1	1
Tumor size	T0	1	0	1	1
	T1	207	50	38	47
	T2	157	38	26	32
	T3	19	5	10	12
	T4	11	3	3	4
	T unknown	16	4	3	4
Lymph node status	N0	234	57	39	48
	N1	162	39	27	33
	N2	7	2	5	6
	N3	0	0	10	12
	N unknown	8	2	0	0
Stage	I	170	41	25	31
	II	206	50	36	44
	III	35	9	19	23
	Unknown	0	0	1	1
Grade	1	53	13	0	0
	2	149	36	7	9
	3	132	32	70	86
	Unknown	77	19	4	5
ER status	ER^+^	276	67	0	0
	ER^-^	131	32	81	100
	ER unknown	4	1	0	0
HER2 status	HER2^+^	62	15	0	0
	HER2^-^	333	81	81	100
	HER2 unknown	16	4	0	0
Recurrence		108	26	55	68
Death		58	14	12	15

Data include 12 patients from the larger Roswell Park Cancer Institute (RPCI) validation study. See Results section for details. CCIH, Clearview Cancer Institute in Huntsville; ER, estrogen receptor.

**Table 2:** Clinical and pathologic characteristics of the Roswell Park Cancer Institute neoadjuvant cohort

Roswell Park Cancer Institute neoadjuvant			
		Number	Percentage
Total patients		23	100
Age	< 50 years	13	57
	≥ 50 years	10	43
	Unknown	0	0
Tumor size	T0	3	13
	T1	6	26
	T2	7	30
	T3	7	30
	T4	0	0
	T unknown	0	0
Node status	N0	7	30
	N1	5	22
	N2	5	22
	N3	6	26
	N unknown	0	0
Stage	I	0	0
	II	10	43
	III	13	57
	Unknown	0	0
ER status	ER^+^	0	0
	ER^-^	23	100
	ER unknown	0	0
Grade	1	0	0
	2	2	9
	3	17	74
	Unknown	4	17
HER2 status	HER2^+^	0	0
	HER2^-^	23	100
	HER2 unknown	0	0
Recurrence		11	48
Death		9	39

ER, estrogen receptor.

**Table 3:** Chemotherapy regimens used in the Clearview Cancer Institute in Huntsville and Roswell Park Cancer Institute cohorts

Regimen	Number
CCIH	
AC	44
CAF	22
AC + pT	43
AC + dT	11
A + dT	7
CAF + pT	2
AC + pT + dT	2
CMF	72
	
RPCI	
AC	17
AC + pT	33
AC + dT	12
pT^a^	1
P + pT^a^	1

Since the Roswell Park Cancer Institute (RPCI) cohort had more standardized treatment (97% had AC or AC + T), the two patients who did not receive AC as part of their regimen were filtered from the analysis. ^a^Patients not included in the analysis. A, doxorubicin; AC, doxorubicin + cyclophosphamide; CAF, cyclophosphamide, doxorubicin, and 5-fluorouracil; CCIH, Clearview Cancer Institute in Huntsville; CMF, cyclophosphamide, methotrexate, and 5-fluorouracil; dT, docetaxel; P, carboplatin. pT, paclitaxel; T, taxane (paclitaxel or docetaxel).

### Tissue arrays, immunohistochemistry, and scoring

**Figure 1:**
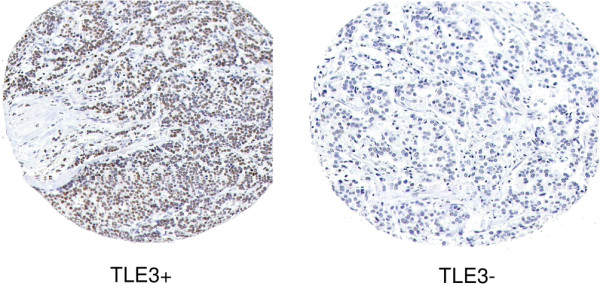
TLE3^+ ^immunohistochemical staining gives a stronger pattern in the nucleus of breast carcinoma cells than TLE3^- ^immunohistochemical staining does.

Duplicate CCIH cohort TMA blocks that each contained single 0.6-mm cores sampled from representative paraffin blocks from each patient were constructed, whereas the RPCI TMA was constructed using duplicate cores from each patient in a single block. TMA sections were dehydrated by submersion in xylene three times for 10 minutes each to remove paraffin, rinsed three times in 100% ethanol and two times in 95% ethanol, and boiled in a microwave for 11 minutes in 10 μM buffered citrate (pH 6.0). Slides were allowed to cool to room temperature and were rinsed in distilled water and then in phosphate-buffered saline (PBS). Slides were dipped in 0.03% hydrogen peroxide, rinsed with PBS, and stained using antibody diluted to appropriate titer in Dako Diluent (DakoCytomation, Glostrup, Denmark) for one hour at room temperature. As a control for staining quality and to select titer, candidate dilutions were first tested on a small 'titer' tissue array that contained positive and negative breast cancer cases and tumor-derived cell lines suspended in paraffin. IHC analysis for TLE3 was performed using a polyclonal affinity-purified antibody at a titer of 1:200. Secondary antibody was applied for 1 hour and staining was visualized using the DakoCytomation Envision staining kit in accordance with the instructions of the manufacturer. A case was scored as positive if greater than 30% of the tumor cell nuclei showed staining, regardless of the staining intensity. Cases without evaluable tumor on any available specimen were removed from the study. Staining with TLE3 in breast cohorts showed differences in a fraction of cases stained as well as variation in intensity of staining. However, there was a subjectively clear delineation between sporadic staining of nuclei and near-homogenous staining (staining of all nuclei). An example of IHC staining for TLE3 is shown in Figure 1. A variety of tumors with expected positives and negatives served as controls. A 30% cutoff was selected to formalize this subjective impression without consideration of a relationship to clinical outcome when the initial cohort was evaluated. This staining rule was prospectively designated prior to staining the validation cohort. Disagreements between replicates were reviewed using an online image database and 'consensus' staining scores assigned prior to clinical data cross-referencing. Staining of TLE3 was performed on tissue obtained from the surgical resection in the discovery and validation cohorts. In the subset of cases which received neoadjuvant therapy at RPCI and in which no tissue was available in the final surgical specimen (pCR), TLE3 staining was performed on the diagnostic core biopsy obtained prior to treatment.

### Statistical considerations

The TLE3 biomarker was identified as a candidate predictive marker for taxane response in a large survey of biomarkers in the CCIH cohort and therefore a correction for multiple testing would be required in order to assess the significance of its association with outcome in the CCIH discovery study. The reported *P *values reflect significance for uncorrected associations. The study at RPCI was prospectively assembled to test the hypothesis from the CCIH cohort. The RPCI taxane arm (n = 45) was 80% powered to test the association with outcome found in the CCIH taxane-treated arm, whereas the AC arm (n = 17) was 36% powered to test an association with outcome if it existed. Independence of TLE3 staining from other clinical and pathologic prognostic parameters was tested by placing TLE3 in a Cox proportional hazards model with each variable in a bivariable model. Tumor response was calculated as the difference between clinical tumor size estimated prior to neoadjuvant treatment subtracted from that estimated after treatment. The relationship between recurrence within 5 years of diagnosis and tumor response was assessed using S-plus software (Tibco Software Inc., Palo Alto, CA, USA) by both a Cox proportional hazards and logistic regression model. The coefficients generated by the latter were used in Excel (Microsoft Corporation, Redmond, WA, USA) to generate the logistic regression curve relating probability of recurrence with tumor response. The predicted association between TLE3 staining and stronger response to treatment was confirmed using a Student *t *test with significance tested using a one-sided *P *value. All univariate and bivariate Cox proportional hazard ratios (HRs), the Fisher exact test, and associated *P *values were calculated using S-plus software.

## Results

### Discovery of TLE3 as a candidate taxane predictive biomarker

**Figure 2:**
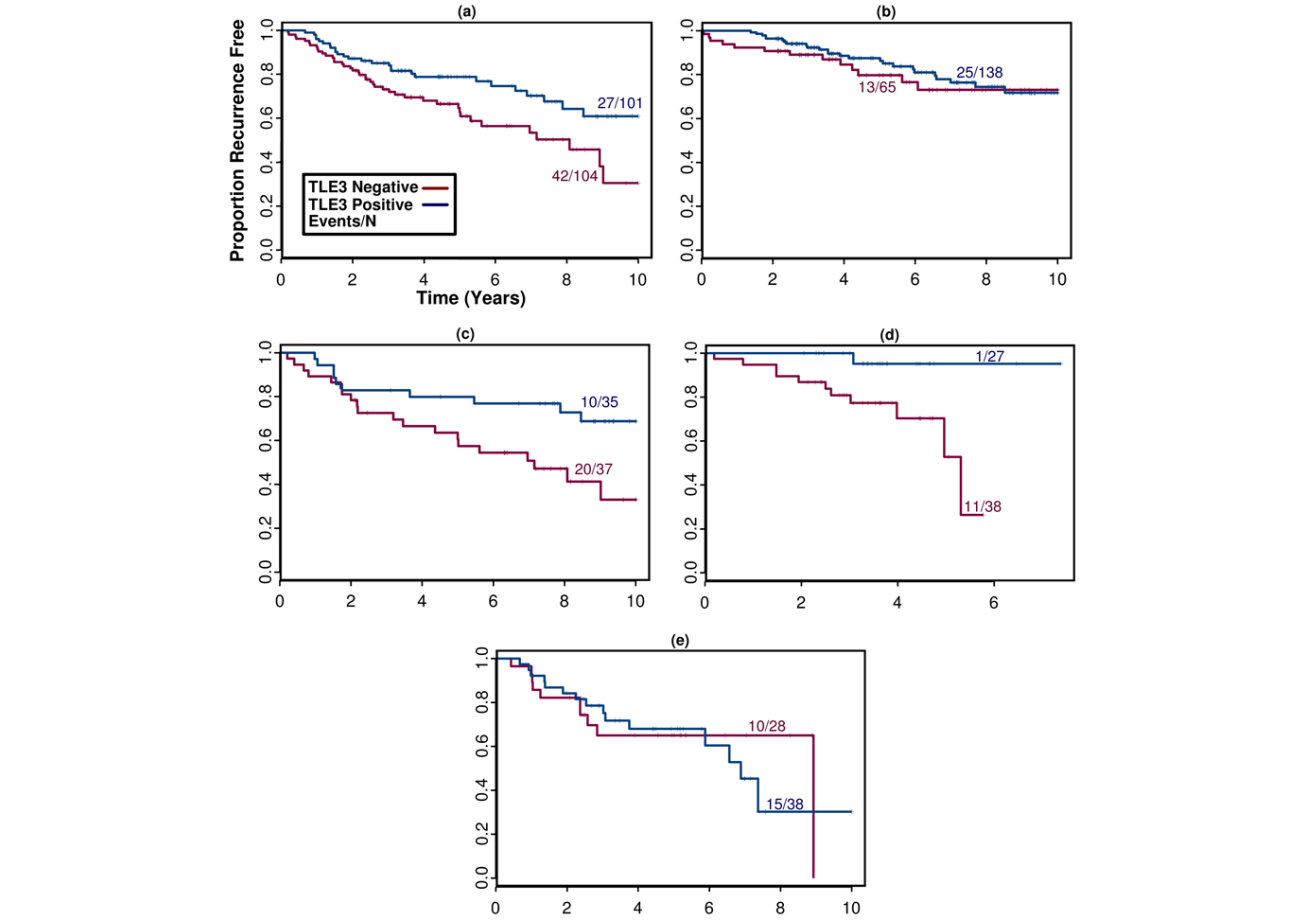
Kaplan-Meier plots in the Clearview Cancer Institute in Huntsville 'discovery' cohort. Patients in this cohort were treated with **(a) **any adjuvant chemotherapy regimen, **(b) **no adjuvant chemotherapy, **(c) **CMF, **(d) **AC (± F) ± taxane, **(e) **AC (± F) but not a taxane. AC, doxorubicin + cyclophosphamide; CMF, cyclophosphamide, methotrexate, and 5-fluorouracil; F, 5-fluorouracil.

Our group has undertaken a large-scale project to screen commercially available and gene expression-targeted novel antisera for utility in classifying breast cancer and identifying individual reagents or combinations of reagents with clinical utility as biomarkers. The identification of TLE3 as a candidate predictive marker for chemotherapy response came from exploration of an existing dataset of IHC stains performed on a single-institution cohort assembled at the CCIH [8]. This cohort of 411 patient samples was stained with over 100 selected antisera (out of approximately 1,000 candidates screened as candidate breast cancer classifiers on non-clinical breast cancer TMAs). TLE3 protein expression was found to be associated with lower risk of recurrence in patients treated with cytotoxic chemotherapy (HR = 0.5, *P *= 0.013). However, no association between TLE3 expression and recurrence was observed in patients treated without adjuvant chemotherapy (HR = 0.8, *P *= 0.49) (Figure 2a,b). Upon further exploration of the data, TLE3 expression was found to be associated with an improved outcome in those patients who received adjuvant cytotoxic chemotherapy with CMF (Figure 2c) or regimens containing AC plus a taxane (n = 65, HR = 0.1, *P *< 0.04) (Figure 2d). No association between TLE3 expression and outcome was seen in patients treated with regimens that contained AC alone (for example, AC or CAF, n = 66, HR = 1.03, *P *= 0.97) (Figure 2e). Ki67/MIB1 staining, a marker of proliferation status, was not associated with chemotherapy response or TLE3 expression (data not shown). In bivariable analysis of taxane-treated patients, TLE3 staining was significantly associated with outcome in the presence of all available clinical and pathologic parameters, including ER and HER2 status (Table 4).

**Table 4:** Bivariable analysis of TLE3 with clinical and pathologic prognosticators

	CCIH				RPCI			
	TLE3		Clinical variable		TLE3		Clinical variable	
Variable	HR	*P *value	HR	*P *value	HR	*P *value	HR	*P *value
Age	0.114	0.038	1.002	0.94	0.15	0.02	0.98	0.52
Tumor size	0.125	0.047	1.026	0.91	0.13	0.01	1.18	0.07
Node status	0.123	0.045	0.423	0.22	0.14	0.02	1.04	0.21
Stage	0.010	0.035	2.24	0.25	0.15	0.02	1.58	0.25
Grade	0.125	0.048	0.95	0.83	0.15	0.02	0.61	0.29
Ki67	0.118	0.041	0.82	0.74	0.09	0.03	0.53	0.45
EGFR	0.09	0.028	2.1	0.37	0.15	0.02	0.63	0.66
ER	0.116	0.042	0.956	0.94	NA	NA	NA	NA
HER2	0.099	0.028	5.92	0.01	NA	NA	NA	NA

TLE3 was tested for independence in the Clearview Cancer Institute in Huntsville (CCIH) and Roswell Park Cancer Institute (RPCI) triple-negative cohorts with a range of standard clinical prognosticators. Independence was tested by placing TLE3 in a Cox proportional hazards model with each of the above variables. In all equations, TLE3 remained a significantly independent predictor of taxane sensitivity (*P *values ranging from 0.008 to 0.048, hazard ratios [HRs] from 0.05 to 0.29). In the validation cohort, tumor size, number of metastatic nodes, and stage remain independent prognosticators to TLE3^+^, while age trends in the right direction. Grade and Ki67 are not independent prognosticators; this is likely due to the fact that the cohort consists almost entirely of high-proliferative tumors. EGFR, epidermal growth factor receptor; ER, estrogen receptor; NA, not applicable.

### Independent validation in 'triple-negative' breast tumors

**Figure 3:**
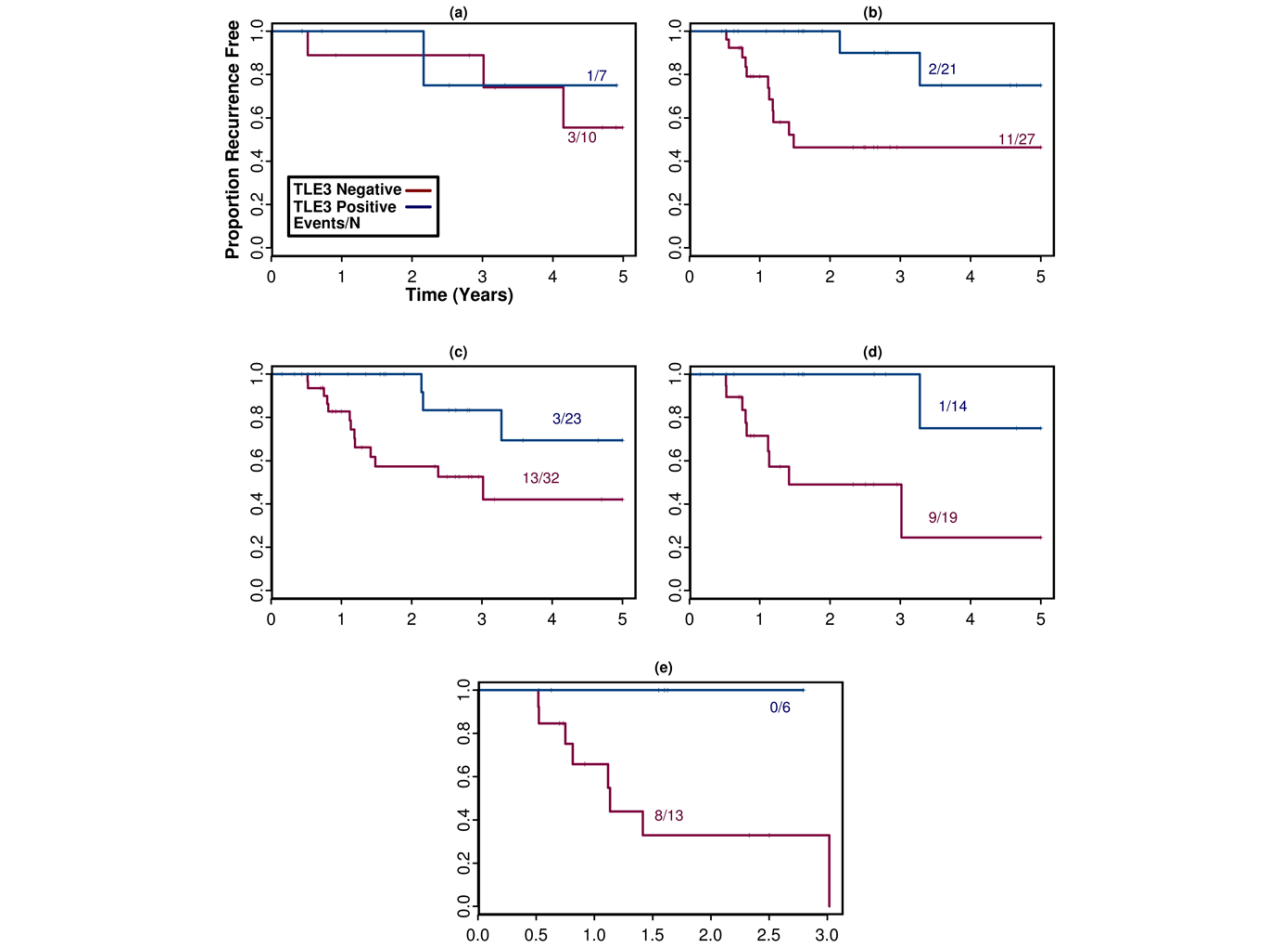
Kaplan-Meier plots depicting recurrence in the Roswell Park Cancer Institute validation 'triple-negative' cohort. Patients in this cohort were treated with **(a) **doxorubicin + cyclophosphamide (AC) without a taxane or **(b) **AC plus a taxane. Patients treated with AC plus a taxane were furthered filtered to **(c) **stage II or greater, **(d) **stage IIB or greater, or **(e) **stage III or greater.

Since a large number of antibodies were used to stain the CCIH cohort, the significance of the association between TLE3 expression and response to taxane therapy is confounded by multiple-hypothesis testing. To independently validate the association between TLE3 staining and outcome among taxane-treated patients, we assembled a new 81-patient TN tissue array cohort using archived paraffin blocks of surgical specimens from RPCI. In this prospectively designed study, TLE3 staining was again associated with favorable outcome but only when patients were treated with an AC regimen that included a taxane (n = 56, HR = 0.15, *P *= 0.018) as opposed to AC alone (n = 17, HR = 0.76, *P *= 0.97) (Figure 3a,b). The association with outcome in taxane-treated patients was present regardless of stage at diagnosis (Figure 3c–e). In bivariable analysis with TLE3^+^, only tumor size trended toward an independent association with outcome in taxane-treated patients whereas age, node status, pathologic grade, and Ki67 and epidermal growth factor receptor (EGFR) expression were not significant (Table 3).

**Figure 4:**
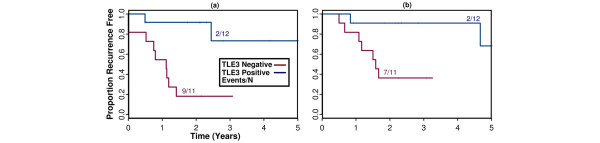
Kaplan-Meier plots depicting **(a)** 5-year recurrence and **(b)** 5-year overall survival among the patients treated neoadjuvantly with doxorubicin + cyclophosphamide plus a taxane in the Roswell Park Cancer Institute triple-negative cohort.

**Figure 5:**
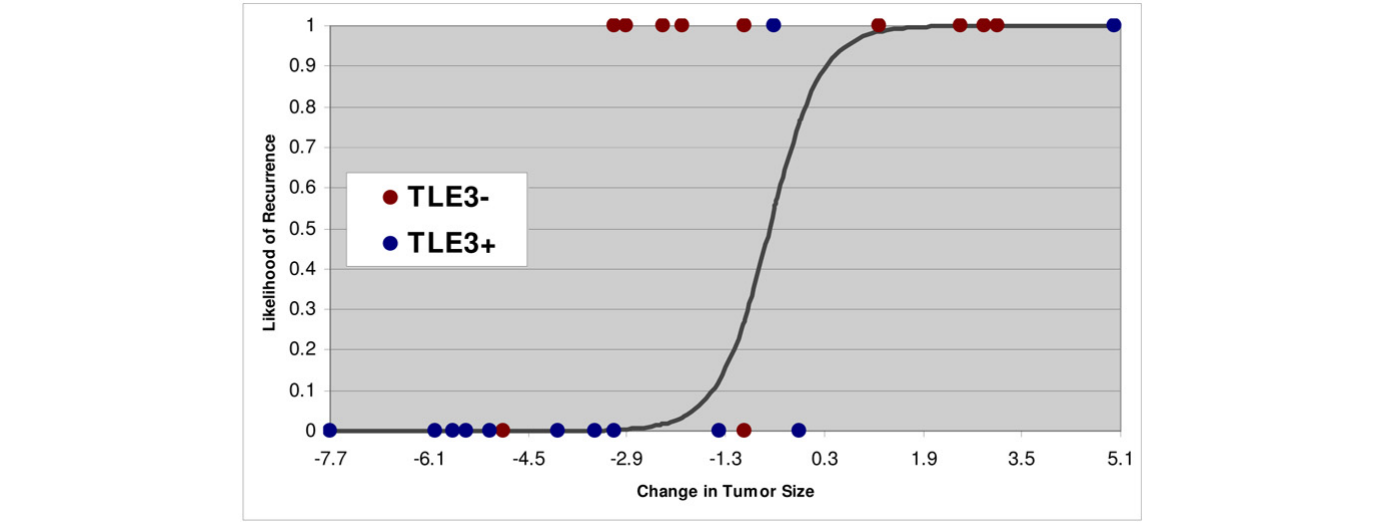
Logistic regression showing the relationship between the change in tumor size in centimeters (measured before and after neoadjuvant chemotherapy) and the likelihood of recurrence. The circles represent the actual patients, color-coded according to TLE3 expression and plotted at either 0 (recurrence-free) or 1 (recurred). A large reduction in tumor size from neodadjuvant therapy correlated with lack of recurrence and was enriched for TLE3^+ ^patients (bottom left corner).

Although this study was designed primarily to explore the utility of TLE3 as a candidate biomarker in the adjuvant setting, we also assembled available patient samples for assessing whether it might be useful in the neoadjuvant setting to predict those patients who would respond to pre-operative taxane treatment. Twelve patients from the original RPCI study who received neoadjuvant taxane therapy were combined with 11 TN patients who were originally excluded from the study because not enough viable tumor tissue was available in the post-neoadjuvant surgical specimen for tissue procurement. Stains were performed on the pre-treatment diagnostic core biopsies for these 11 new patients. In this study of neoadjuvant taxane-treated patients (n = 23), TLE3 expression was also associated with favorable outcome (recurrence: HR = 0.12, *P *= 0.0093; survival: HR = 0.11, *P *= 0.042) (Figure 4). Available archived clinical records and imaging studies were reviewed to approximate the change in tumor size in response to neoadjuvant therapy for 21 of the study patients. Tumor size reduction was highly correlated with likelihood of recurrence at 5 years (HR = 1.56, *P *= 0.0001), and a significant association between staining with TLE3 and decrease in tumor size was confirmed (Figure 5, *t *test one-sided *P *value < 0.04).

## Discussion

In this study of breast cancer patients receiving adjuvant chemotherapy at two independent institutions, TLE3 staining was associated with improved disease-free survival in patients receiving a taxane-containing regimen as opposed to anthracycline without taxane. The CCIH cohort in which the association between taxane treatment and outcome was first discovered was comprised of both ER-expressing and ER^- ^patients. The RPCI TN cohort was prospectively assembled as an independent validation study to test the association between TLE3 staining and taxane sensitivity discovered in the CCIH cohort. The TN cohort was chosen for three reasons: (a) to remove the confounding effects of ER and HER2, (b) taxanes are routinely given as standard of care in this population, and (c) the risk of recurrence is higher in TN breast cancer and therefore a large number of disease progression events were available for analysis. Although patients in the CCIH cohort were treated with variable chemotherapeutic regimens and anti-hormonal treatment for ER^+ ^patients, the association of TLE3 with taxane sensitivity was independent of stage, grade, and ER and HER2 status. In the validation study, in which treatment was standardized to AC or AC + T, the association between TLE3 staining and treatment with a taxane-containing regimen was confirmed and was found to be independent of Ki67/MIB1 and EGFR expression, stage, and grade (the cohort was predominantly high-grade overall). As this cohort was also confirmed to be ER^- ^and HER2^- ^by re-staining, TLE3 is not acting as a surrogate for proliferation, HER2, or ER.

The association of TLE3 with outcome was also apparent in the subset of patients who received neoadjuvant taxanes as part of their regimen. In addition, retrospective review of tumor response to treatment showed a significant association between TLE3 expression and decrease in tumor size after neoadjuvant treatment. Unfortunately, our group was too small to test a significant association between pCR and TLE3 expression. Of the eleven core biopsy samples for which tumor material was apparently completely depleted by neoadjuvant therapy, seven were TLE3^+ ^and only one of these seven ultimately recurred. While these data are exploratory and do not incorporate more recent quantitative methods of measuring tumor response, the possibility of using TLE3 as tool to predict response in the neoadjuvant setting is intriguing and warrants further study in a prospective trial [15].

TLE3 is a transcriptional repressor that appears to be periodically expressed during the M phase of the cell cycle [13,14]. Other TLE family members have been implicated in tumorogenic pathways, and TLE1 when overexpressed leads to lung adenocarcinoma in mice and also has been confirmed as a biomarker able to differentiate synovial sarcomas [10,16,17]. TLE family members have been shown to interact with the Notch pathway members and be phosphorylated by mitogen-activated protein kinase in the nucleus in response to EGFR signaling, potentially modulating Notch pathway signaling [18,19]. More recently, TLE3 was identified in a screen for genes causing estrogen independence in breast cancer cell lines and RNA levels further shown to be associated with progression-free survival in ER-expressing patients treated with tamoxifen as first-line treatment for metastatic disease [20,21]. In this study, TLE3 expression was associated with both methotrexate and taxane sensitivity in the discovery cohort and was confirmed to be associated with taxane sensitivity in the validation cohort. This raises the possibility that, as opposed to being related only to taxane therapy, it may be a candidate marker of sensitivity to cell cycle-targeted cytotoxic therapeutics. By IHC, it is expressed exclusively in the nucleus in a subset of carcinoma cases. Its pattern of expression both within a single tumor and across a large number of tumor cases is distinct from that of classic proliferation markers such as Ki67. Since TLE proteins are known to interact directly with chromatin and chromatin-associated proteins, it may identify cells in a differentiation state particularly sensitive to cell cycle perturbation [22]. Further studies are needed to explore whether TLE3 is only a marker of sensitive cells or whether it can be implicated more directly in sensitizing cells to chemotherapy-mediated cell death.

Taxanes are one of the most active agents against breast cancer and are routinely used in metastatic, adjuvant, and neoadjuvant settings. Results from a number of large randomized trials demonstrate that the use of both paclitaxel and docetaxel can result in improved outcomes in women with breast cancer of all stages [3,4,6,7]. A number of studies have looked at clinical and pathologic parameters that might predict response to taxanes, most notably the candidate biomarker tau, but evidence in support of the use of these biomarkers is still preliminary [23,24]. A recent study by Hayes and colleagues [25] suggested that HER2^+ ^breast tumors may gain increased clinical benefit from treatment with taxanes. However, other studies have shown no association between response to taxanes and HER2 overexpression [26]. Taxane-containing regimens have significant short-term toxicity compared with the conventional anthracycline-containing combinations, particularly related to myelosuppression, peripheral neuropathy, and hypersensitivity reactions. Long-term toxicities from taxane use are still largely unknown. At present, no biomarkers associated with taxane response have been validated to a confidence level that would allow them to be incorporated into standard practice.

The discovery of TLE3 as a candidate biomarker of taxane sensitivity in the CCIH study is confounded by the use of a variety of therapies and exploration of a large number of candidate biomarkers in this hypothesis-generating study. Although the validation study was relatively small and specifically the AC arm was underpowered to test for an interaction between TLE3 and taxane treatment, the confirmation of an association of TLE3 with outcome in taxane-treated patients while showing no association with outcome in patients treated only with anthracylines is consistent with the predictive hypothesis generated in the CCIH study. The consistency of the findings across cohorts and the strength of the association with outcome in predominantly high-grade tumors treated with taxanes in the independent TN validation study support TLE3 as a biomarker of response to taxane therapy. We believe TLE3 should be studied in larger clinical trial populations to further assess its potential as a predictive marker for taxane therapy in breast cancer.

## Conclusions

This study of IHC staining with TLE3 antibody in breast carcinoma and its association with outcome supports the hypothesis that increased TLE3 expression may predict those patients who will respond to taxane therapy. Furthermore, it is remarkable that, in the validation study, TLE3 staining was associated with response to taxane therapy in women with aggressive TN disease in which there is an absence of established molecular targets for targeted therapy. Clearly, the identification of high-quality biomarkers capable of identifying women with a high likelihood of response to taxanes would represent a significant advance in our ability to effectively treat women with breast cancer. Additional study of TLE3 as a candidate biomarker is warranted in larger clinical trial populations to confirm these findings.

## Abbreviations

A: doxorubicin; AC: doxorubicin + cyclophosphamide; C: cyclophosphamide; CAF: cyclophosphamide, doxorubicin, and 5-fluorouracil; CCIH: Clearview Cancer Institute in Huntsville; CMF: cyclophosphamide, methotrexate, and 5-fluorouracil; EGFR: epidermal growth factor receptor; ER: estrogen receptor; F: 5-fluorouracil; HR: hazard ratio; IHC: immunohistochemistry; M: methotrexate; MRI: magnetic resonance imaging; P: carboplatin; PBS: phosphate-buffered saline; pCR: complete pathologic response; PR: progesterone receptor; RPCI: Roswell Park Cancer Institute; T: taxane; TLE: transducin-like enhancer of split; TMA: tissue microarray; TN: triple-negative.

## Competing interests

RAB, BZR, MTS, RSS, and DTR are stockholders in Applied Genomics, Inc. (Huntsville, AL, USA). All other authors declare that they have no competing interests.

## Authors' contributions

SAK drafted the manuscript, participated in study design developed from the dataset at RPCI, and coordinated RPCI participation. DGH created TMAs at RPCI, read IHC for TLE3, and helped to draft the manuscript. NLW was the data manager of the RPCI breast database and designed the database for the RPCI patients. CM helped to prepare the manuscript and to coordinate the data collection from RPCI. HH reviewed slides of neoadjuvant cases. TK read TMAs for EGFR. RAB scored TLE3 TMAs. BZR participated in the statistical analysis. NCE and MTS participated in the design of the study. RSS conceived of the study, performed statistical analysis, and participated in manuscript preparation. DTR was involved in concept development, creation of the study design, manuscript development, and coordination of the study. All authors read and approved the final manuscript.
